# Presence and Relevance of Emerging Microorganisms in Clinical Genitourinary Samples

**DOI:** 10.3390/microorganisms11040915

**Published:** 2023-03-31

**Authors:** Antonio Rosales-Castillo, Manuela Expósito-Ruiz, Miguel Gutiérrez-Soto, José María Navarro-Marí, José Gutiérrez-Fernández

**Affiliations:** 1 Department of Internal Medicine, Virgen de las Nieves University Hospital and Doctoral Program of Clinical Medicine and Public Health, University of Granada, Granada Institute of Biohealth Research (Ibs.), Avda. de las Fuerzas Armadas 2, 18014 Granada, Spain; 2 Biostatistics Unit, Department of Statistics, School of Medicine, University of Granada, 18012 Granada, Spain; 3 Emergency Department, Montilla Hospital, 14550 Cordoba, Spain; 4 Laboratory of Microbiology, Virgen de las Nieves University Hospital, Granada Institute of Biohealth Research (Ibs.), Avda. de las Fuerzas Armadas 2, 18014 Granada, Spain; 5 Department of Microbiology, School of Medicine, University of Granada, Granada Institute of Biohealth Research (Ibs.), Avenida de la Investigación 11, 18016 Granada, Spain

**Keywords:** genital and urinary infection, emerging pathogens, clinical significance

## Abstract

Microorganisms responsible for genitourinary infections increasingly include species other than conventional etiological agents that are of clinical and pathogenic relevance and therapeutic interest. This cross-sectional descriptive study selected samples from clinical genitourinary episodes between January 2016 and December 2019 in which emerging microbiological agents were detected. The patients’ epidemiological characteristics, clinical presentation, antibiotic treatment, and outcome were studied to identify their pathogenic role. The emerging microorganisms most frequently detected in urinary tract infections were *Streptococcus bovis* (58.5%) and *Gardnerella* spp. (23.6%) in females and *S. bovis* (32.3%), *Aerococcus urinae* (18.6%), and *Corynebacterium* spp. (16.9%) in males, while the most frequently detected in genital infections were *S. viridans* (36.4%) in females and *C. glucuronolyticum* (32.2%) and *Gardnerella* spp. (35.6%) in males. All cases in female children were produced by *S. bovis.* Symptomatic episodes were more frequent with *Aerococcus* spp. and *S. bovis* and the presence of leukocytosis more frequent with *Aerococcus* spp. Quinolones and doxycycline were most often prescribed antibiotics for genital infections and quinolones and amoxicillin-clavulanic acid for urinary infections. Urinary infection by *Aerococcus* spp. was more frequent in males of advanced age, *Corynebacterium* spp. was more frequent in permanent vesical catheter carriers, and episodes of asymptomatic bacteriuria by *Gardnerella* spp. were more frequent in patients with kidney transplant and chronic consumers of corticosteroid therapy. *Lactobacillus* spp. should be considered in urinary infections of patients of advanced age and with a previous antibiotic load. Genital infection by *Gardnerella* spp. was significantly associated with a history of risky sexual relations.

## 1. Introduction

Infection is defined as the invasion of a host organism’s bodily tissues by disease-causing organisms and can result from the interplay between pathogens and the defenses of the hosts they infect [1]. Urinary tract infection (UTI) is the second most frequent infection in humans after respiratory tract infection, affecting 150 million people a year worldwide and posing a serious public health problem [2]. It mainly appears in healthy women with no underlying disease or functional or structural urinary tract anomaly. Recent sexual activity is considered the most important risk factor for UTI, which may explain its higher incidence between the ages of 18 and 40 years; it has been estimated that around 50–60% of adult women will have at least one UTI episode during their lifetime [3]. Most cases are non-complicated UTIs, although these can have a major health impact given possible relapses and the potential progression to pyelonephritis or sepsis, damaging the kidney and even causing preterm delivery in pregnant women [4]. UTIs can be classified as low (acute cystitis, urethritis, and prostatitis), high (pyelonephritis and pyonephrosis), or, according to the absence or presence of risk factors (childhood age, male sex, pregnancy, presence of urinary catheter and/or recurrent, obstructive, functional, or structural disorders), non-complicated.

Etiologically, Escherichia coli is responsible for 80–85% of UTI cases, with the remainder produced by *Proteus* spp., *Klebsiella* spp., *Serratia* spp., *Pseudomonas* spp., *Staphylococcus* spp., *Enterococcus* spp., *Enterobacter* spp., *Citrobacter* spp., *Acinetobacter* spp., or *Candida* spp. The etiology of UTI can be modified by various risk factors, including the age, sex, and institutionalization of patients, the presence of diabetes, spinal cord lesions, urinary catheterization, and previous urinary tract instrumentation and/or the receipt of antibiotic [3]. For their part, genital infections are classified as vulvovaginal and cervical in females according to their localization [5] and are most frequently produced by candidiasis, trichomoniasis, or anaerobic bacteria associated with vaginal dysbiosis, such as *G. vaginalis*, *G. swidsinskii*, or *G. piotii* [6,7]. The most prevalent entities in males are urethritis, balanitis, and chronic prostatitis, considered responsible for 6–15% of cases of male infertility and most often caused by Chlamydia trachomatis and Neisseria gonorrhoeae. 

However, other pathogens are emerging as responsible for these diseases in certain clinical settings. These microorganisms may have been overlooked or poorly classified due to the lack of distinctive phenotypic criteria, the consideration of their significant growth as contamination by microbiota, and failure in their detection by standard methods, sometimes because of their slow growth and the need for nutritionally demanding culture media [8,9]. Advances in microbiological techniques have revealed new scenarios of clinical and microbiological relevance and increased the detection of these pathogens, including *Corynebacterium* spp., *Aerococcus* spp., *Actinotignum* spp., *Actinobaculum massiliense*, *Actinomyces turicensis*, *Alloscardovia omnicolens*, *Aeromonas hydrophila*, *Eikenella corrodens*, *Lactobacillus* spp., *Streptococcus bovis* group (SBG), *pneumoniae*, and *viridans* group (SVG), *Leptotrichia trevisanii*, *Facklamia* spp., *Pasteurella* spp., *Neisseria meningitidis*, and *Gardnerella* spp. [10].

The objective of this study was to analyze the clinical characteristics of episodes associated with the genitourinary presence of these emerging microorganisms, including clinical manifestations, analytical findings, risk factors, comorbidities, outcomes, and therapeutic approaches, with the aim of facilitating early recognition and clinical suspicion of these etiologic agents and improving clinical treatments and the prognosis.

## 2. Material and Methods 

### 2.1. Study Design

The study included 312 clinical samples with the single isolation of an emerging microorganism responsible for genitourinary disease received by the microbiology laboratory of Virgen de las Nieves University Hospital (Granada, Southern Spain) between January 2016 and December 2019; 89 of samples corresponded to genital infection episodes and 223 to urinary infection episodes. Samples were from patients attended in the Emergency (General and Pediatric), Urology, and Infectious Diseases Departments, which cover the populations of Granada city and its metropolitan area. Samples came from different episodes (≥6 weeks after any previous episode), and all were processed in accordance with the established laboratory protocol [10].

### 2.2. Microbiological Procedures

Samples from patients with suspicion of UTI were collected from mid-stream micturition, permanent catheterization, provisional catheterization, pediatric urine collection bag, or nephrostomy catheter under anti-contamination conditions. They were processed using a 1-μL calibrated loop and UriSelect 4 chromogenic culture medium (Bio-Rad, Barcelona, Spain) incubated for 24 h at 37 °C. In samples from the Nephrology Department, a sheep blood agar plate (Becton-Dickinson, Madrid, Spain) was added and incubated in CO_2_. Breakpoints for colony growth were: Negative (<10,000 UFC/mL and <1000 UFC/mL in urine from provisional catheterization); Positive (>100,000 UFC/mL of one or two uropathogens, or 10,000–100,000 of a single uropathogens, or, in urine from provisional catheterization, >10,000 UFC/mL of one or two uropathogens or 1000–10,000 UFC/mL of a single uropathogen); or Mixed (>10,000 UFC/mL of more than two uropathogens). Real-time multiplex PCR was used in semen, glans, endocervical, genital ulcer, urethral, and rectal exudate samples from patients with suspicion of genital infection to determine the presence of *Chlamydia trachomatis*, *Neisseria gonorrhoeae*, *Trichomonas vaginalis* (BD MAX CT/GC/TV BD, Franklin Lakes, NJ, USA), *Mycoplasma genitalium*, *Mycoplasma hominis*, and *Ureaplasma urealyticum* (BD MAX System, BioGX DNA, 350-011-A-MAX, Amsterdam, the Netherlands). DNA hybridization tests and BD MicroProbe Processor were used for the detection of *Gardnerella* spp., *Candida* spp., and *Trichomonas* (BD AFFIRM VPIII, BD, Madrid, Spain) in samples of vulvovaginal exudates, which were also seeded on blood agar (Becton-Dickinson), chocolate agar (Becton-Dickinson), Martin-Lewis agar (Becton-Dickinson) media for *N. gonorrhoeae* and on chromogenic agar medium for *Candida* spp. (BIO-RAD). A significant result was defined by monomicrobial and abundant growth (to third seeding area) of an opportunistic pathogen or the presence of a strict pathogen. Infection with *M. hominis* or *U. urealyticum* was recorded when a value of Ct ≤ 30 was obtained. MALDI-TOF mass spectrometry (Biotyper, Brucker Daltonics, Billerica, MA, USA) and/or MicroScan Walkaway (Beckman-Coulter, Brea, CAL) were used for the optimal identification of isolated microorganisms. All microorganisms were identified by MALDI-TOF mass spectrometry except for SBG and *Aeromonas hydrophila*, which were identified using MicroScan Walkaway.

### 2.3. Study Variables

Clinical and epidemiological data were gathered from electronic clinical records on the sex, age, type of sample, microorganism isolated, presence of immunosuppression (due to solid organ transplantation, active neoplasm, chronic corticoid consumption, hemodialysis, diabetes mellitus, infection by human immunodeficiency virus [HIV]), previous risky sexual relations, associated clinical manifestations or asymptomatic episode, pregnancy, therapeutic decision and antibiotic treatment, resolution/relapse, and related mortality. Analytical data: presence of leukocytosis (>12,000/mm^3^), C reactive protein (CRP), and radiological findings (imaging test and any alterations) were also collected. Pathological urine analysis was defined by the presence in urinary sediment of ≥5 leukocytes per high-power field (400X), accompanied or not by nitrites, proteins, or red blood cells. 

### 2.4. Statistical Analysis

In the statistical analysis, absolute (n) and relative (%) frequencies were calculated for qualitative variables and medians with interquartile range (IQR) for continuous variables. The Kolmogorov-Smirnov test was then applied to check the normality of variable distribution. The relationship of microorganism type with epidemiological characteristics, treatment, and clinical outcome was studied by constructing contingency tables to calculate percentages of the variables for each microorganism. The independence hypothesis was tested by using Pearson’s chi-square test or, when applicability conditions were not met (≤20% of expected frequencies < 5), Fisher’s exact test. When Pearson’s chi-square test showed statistical significance (<0.05), corrected typified residues were analyzed to identify the box in the table responsible for the difference. Quantitative variables were analyzed with the non-parametric Kruskall-Wallis test and 2-by-2 comparisons with the Mann-Whitney U test (corrected significance level of 0.005). SPSS 19 was used for data analyses (IBM SPSS, Armonk, NY, USA). 

### 2.5. Ethical Considerations

The study protocol was performed in agreement with the Helsinki Declaration and ethical epidemiological research principles. The study was non-interventionist and involved no change to routine procedures, meeting conditions for exemption from the need for informed consent. The biological material was used solely for the standard diagnosis of urogenital tract infections as prescribed by attending physicians. The protocol for sampling and routine diagnostic procedures was not modified in any way. Data derived from completely anonymous database in which patients were substituted by the infectious episodes (≥6 weeks after previous episode). The permission to access and utilize data was granted by the Clinical Microbiology Management Unit of Virgen de las Nieves Hospital in Granada, Spain. This study was approved by the Ethics and Human Research Committee (Internal code: 2538-N-21).

## 3. Results

Supplementary Table S1 lists the episodes of genital and urinary infections and results for the study variables. Results were first divided between genital infection and urinary infection groups, and each group was then sub-divided into male and female adults and children (<14 years). Clinical episodes associated with immunosuppressive conditions are also reported.

### 3.1. Genital Infection

Out of the 89 samples corresponding to genital infection episodes, 24 (27%) were from females and 65 (73%) from males. Among the 24 females, 22 (91.7%) were adults and 2 (8.3%) children (<14 years). Among the 65 males, 58 (89.2%) were adults and 7 (10.8%) children.

The mean (standard deviation {SD}) age of the 22 female adults was 36.7 (11.9) years, and all had a Charlson index of 0 except for one woman (6, metastatic neoplasm). The localization of samples from females was endocervical in 16/24 (66.7%); genital ulcer in 4/24 (16.7%); vaginal in 3/24 (12.5%), and vulvar in 1/24 (4.2%). Table 1 exhibits the results obtained for epidemiological and clinical variables. There were four episodes (18.2%) of potentially severe pelvic inflammatory disease (PID) produced by *A. massiliense*, *E. corrodens*, SVG, and *L. trevisanii*, respectively. Supplementary Table S2 lists microorganisms isolated in this group and their frequency. Antibiotic treatment was prescribed in 19/22 episodes (86.4%), most frequently with doxycycline + metronidazole (2/19; 10.5%), ampicillin + gentamicin (2/19; 10.5%), and oral metronidazole (2/19; 10.5%). Only two episodes corresponded to female children (2/24; 8.3%), whose samples were from a genital localization and endocervical ulcer, respectively, detecting isolates of *A. schaali* and *A. turicensis*. Both girls had symptoms (100% vaginal pruritus, 50% dysuria, 50% abdominal pain) that were resolved by empirical treatment.

The mean (SD) age of the 58 male adults was 42.8 (16.7) years, and their mean Charlson index was 0.7 (1.7). The origins of samples from adult males were semen (32/58; 55.2%), urethral exudate (20/58; 34.5%); balanopreputial exudate (4/48; 8.3%); rectal exudate (1/58; 1.7%), or genital ulcer (1/58; 1.7%). Table 2 displays their clinical and epidemiological characteristics. Supplementary Table S3 lists microorganisms isolated in this group and their frequency. Antibiotic treatment was prescribed for 40/58 episodes (69%), most frequently with doxycycline (14/40; 35%). The mean (SD) age of the seven male children was 5.6 (4) years, and the origin of their samples was balanopreputial exudate (6/7; 85.7%) or genital ulcer (1/7; 14.3%). A different isolate was detected in each sample, i.e., SVG, *A. urinae*, *A. schaali*, *F. hominis*, *A. sanguinis*, *C. amycolatum*, and *Aeromonas hydrophila*; All cases were symptomatic, including balanitis (5/7; 71.4%), dysuria (2/7; 28.6%), and/or urethral exudate (2/7; 28.6%). All cases were fully resolved after empirical treatment with amoxicillin/clavulanic acid (4/7; 57.1%), topical mupirocin (2/7; 28.6%), or azithromycin (1/7; 14.3%).

### 3.2. Urinary Infection 

Among the 223 urinary infection samples, 77/223 (34.5%) were from males and 146/223 (65.5%) from females. Among the 146 samples from females, 131 (89.7%) were from adults and 15 (10.3%) from children. Among the 77 samples from males, 73 (94.8%) were from adults and 4 (5.2%) from children.

The mean (SD) age of female adults was 60.8 (18.9) years, and their mean Charlson index was 2.25 (1.6). Table 3 exhibits the clinical and epidemiological characteristics of their urinary infection episodes. Clinical situations associated with immunosuppression were rheumatoid arthritis (1/131; 0.8%), liver cirrhosis (1/131; 0.8%), hemodialysis (4/131; 3.2%), hematologic neoplasm (1/131; 0.8%), and solid organ neoplasm (15/131; 12%; 13 under active treatment). Supplementary Table S4 lists microorganisms isolated in this group and their frequency. Nine concomitant isolates were detected, including eight (88.9%) corresponding to *E. coli*. Urinary tract ultrasound was performed in 18/131 (13.7%) of the women. Nephro-urological disorders were detected in 14/131 (10.7%), including renal polycystosis (6/14; 42.9%), renal lithiasis (4/14; 28.6%), acute pyelonephritis (2/14; 14.3%), neoformation (1/14; 7.1%), and pyelocaliceal ectasia (1/15; 7.1%). Antibiotic treatment was prescribed in 77 of the 131 women (58.8%), most frequently cefuroxime (11/77; 14.3%) and amoxicillin/clavulanic acid (10/77; 13%). The mean (SD) age of the 15 female children was 2.3 (2.9) years, and only one was immunosuppressed (hematologic neoplasm). All isolates (15/15; 100%) corresponded to SBG. No nephro-urological disorders were detected. Urine analysis was performed in 12/15 (80%) of the female children and was pathological in 4/12 (33.3%). Associated symptoms were described in 13/15 (86.7%), being the presence of fever in 11/15 (73.3%). Other reported symptoms were abdominal pain (2/15; 13.3%) and dysuria (1/15; 6.7%). CRP was measured in seven (46.7%) of the female children, being altered in three (42.9%), and leukocytosis was reported in two (13.3%). Full resolution was obtained by antibiotic treatment, most frequently with cefixime and amoxicillin, administered to five of the female children (33.3%).

The mean (SD) age of the 73 male adults was 63.5 (16.9) years, and their mean Charlson index was 2.25 (1.8). Table 3 displays their epidemiological and clinical characteristics. Thirty-one men (42.5%) were immunodepressed due to liver transplant (2/31; 6.5%), kidney transplant (22/31; 71%), liver cirrhosis (3/73; 4.1%), hemodialysis (2/73; 6.5%), or active chemotherapy (2/73; 6.5%). All those receiving chronic corticosteroid therapy (21/73; 28.8%) and immunosuppressants (21/73; 28.8%) were transplantation patients. Nephro-urological disorders were observed in 17/73 (23.3%), including benign prostate hyperplasia (6/17; 35.3%), renal lithiasis (4/17; 23.5%), renal polycystosis (3/17; 17.6%), urethral stenosis (2/17; 11.8%), and vesical neoformation (2/17; 11.8%). Kidney and urinary tract ultrasound were performed during the episode in 15/73 cases (20.5%). Antibiotic treatment was prescribed in 49/73 (67.1%), most frequently third-generation cephalosporin (11/49; 22.4%), quinolone (11/49; 22.4%) and amoxicillin/clavulanic acid (10/49; 20.4%). Supplementary Table S5 lists microorganisms isolated in this group and their frequency. Four urinary infection episodes were recorded in the male children, whose mean (SD) age was 4.6 (4.6) years; they corresponded to SBG (*n* = 2), *A. urinae* (*n* = 1), and *A. sanguinicola* (*n* = 1). Symptoms were reported in 3/4 (75%) episodes, including fever (2/4; 50%), abdominal pain (2/4; 50%), and dysuria (1/4; 25%). Urine analysis results were pathological in 2/4 (50%) episodes, and CRP was elevated in 2/4 (50%). Half of these cases were successfully treated with fosfomycin/trometamol and cefixime.

Almost all (66/68; 97%) cases of transplantation were for kidneys. All episodes in transplantation patients were for suspicion of urinary infection or to rule it out, and the most frequently isolated microorganisms were *Gardnerella* spp. (28/68; 41.2%) and SBG (13/68; 19.1%). Urine analysis was performed in 41/68 (60.3%) cases and was pathological in 17/41 (41.5%). Although only 9 (13.2%) of these episodes were symptomatic, antibiotic treatment was prescribed in 27 (39.7%). Besides the patients with solid organ transplants, immunosuppression was recorded in 39 patients due to HIV (6/39; 15.4%), metastatic solid neoplasm (20/39; 51.3%), hematologic neoplasm (2/39; 5.1%), hemodialysis (6/39; 15.4%), liver cirrhosis (5/39; 12.8%), or rheumatoid arthritis (1/39; 2.6%). Nine episodes were for genital infection (6 related to HIV, 2 to solid neoplasms, and 1 to liver cirrhosis) and thirty for urinary infection. Six of the nine genital episodes (66.7%) were symptomatic, and eight (88.9%) were treated with antibiotherapy. Isolates corresponded to SBG in 21 (70%) of the 30 urinary episodes, 17 (56.7%) of which were symptomatic. Urine analyses were conducted in 25 cases, and the result was pathological in 21 (84%). Antibiotic treatment was prescribed in 23/30 (76.7%) of these patients.

Microorganisms were grouped into five main groups for the statistical analysis: *Aerococcus* spp. (*A. sanguinicola*, *A. christensenii*, *A. urinae*, and *A. viridans*); *Gardnerella* spp., *SBG*, *Corynebacterium* spp. (*C. amycolatum*, *C. aurimucosum*, *C. glucurunolyticum*, *C. jeikeium*, *C. minutissimum*, *C. striatum*, and *C. urealyticum*), and Other (including *Actinotignum* spp., *Actinobaculum massiliense*, *Actinomyces turicensis*, *A. hidrophyla*, *Alloscardovia ominocollens*, *Eikenella corrodens*, *Facklamia hominis*, *Lactobacillus*, *Leptotrichia trevisani*, *Moraxella osloensis*, *Neisseria meningitidis*, *Pasteurella bettyae*, *S. grupo viridans*, and *Streptococcus pneumoniae*). The median age was significantly higher (*p* < 0.001) for the *Aerococcus* spp. group (77 yrs) than for the *Corynebacterium* spp. (50 yrs, 32.5–67.5); *Gardnerella* spp. (44 yrs, 29–52), SBG (56 yrs, 39–73), and *Other* (50 yrs, 30.5–66.5) groups. Associated episodes did not significantly differ in the Charlson index of patients.

The type of microorganism was significantly associated with the sex of patients (*p* < 0.001), observing a significantly higher percentage of males with episodes produced by *Aerococcus* spp. (21; 61.8%) and *Corynebacterium* spp. (36; 81.8%) than with episodes produced by *Gardnerella* spp. (28; 47.5%), SBG (22; 23.4%), or Other (35; 43.2%) groups. In relation to immunosuppressive factors, the percentage of solid organ transplantation patients was significantly higher in episodes of *Gardnerella* spp. (28; 47.5%) and significantly lower in episodes of SBG (13; 13.8%) (*p* < 0.001). The percentage of patients receiving corticosteroid therapy was also significantly higher in episodes of *Gardnerella* spp. (25; 42.4%) (*p* < 0.001). A history of risky sexual relations (unprotected, oral/anal/vaginal) was also related to the type of microorganism, finding that a significantly higher (*p* < 0.001) percentage of this type of patient was infected with *Gardnerella* spp. (12; 20.3%). The presence of a permanent catheter was significantly associated with infection by *Corynebacterium* spp. (7; 15.9%) (*p* = 0.0292).

Symptomatic episodes were significantly (*p* = 0.004) more frequent in infections caused by *Aerococcus* spp. (27; 79.4%) and SBG (63; 67%) and asymptomatic episodes more frequent in infections by *Gardnerella* spp. (32; 54.2%) (*p* = 0.004). The presence of balanitis was associated with episodes caused by *Corynebacterium* spp. (3; 6.8%) and *Gardnerella* spp. (3; 5.1%) (*p* = 0.04695); dysuria with episodes by *Aerococcus* spp. (11; 32.4%) and *Gardnerella* spp. (16; 27.1%) (*p* = 0.034); fever with episodes due to *Aerococcus* spp. (14; 41.2%) and SBG (47; 50%) (*p* < 0.001); and leucorrhea with species in the *Other* group (11; 13.6%) (*p* = 0.00026). In relation to analytical parameters, no association was found with CRP elevation, but the presence of leukocytosis was significantly more frequent in infections caused by *Aerococcus* spp. (10; 29.4%) and SBG (24; 25.5%) (*p* = 0.006).

## 4. Discussion

Diagnostic improvements and a more appropriate and ecological prescription of antibiotic treatments have resulted from major advances in clinical microbiology, including the increasingly widespread application of mass spectrometry and molecular techniques as well as the utilization of enriched media for demanding bacteria in prolonged culture [11]. 

UTI may have been underdiagnosed due to the consideration of some emerging microorganisms as contaminants. For instance, *Aerococcus* spp. and SBG can be confused with enterococci and streptococci due to their phenotypic similarities, and the detection of *Corynebacterium* spp. may be limited by their growth characteristics and need for enriched media [8]. It is important to consider factors that may increase the risk of infection by these agents, including the presence of anatomical urinary tract or prostate disorders, diabetes mellitus, permanent vesical catheter, solid organ transplantation, pharmacological immunosuppression, or recent antibiotic consumption (in previous 3 months) [12]. 

In this study, it was observed that genital infections in female adults were produced by a wide range of isolates, most frequently SVG (31.8%) corresponding to *Anginosus* and *Constellatus* species. These microorganisms are part of habitual oral, gastrointestinal, and urogenital microbiota and can potentially cause infectious symptoms with a tendency to abscess formation but rarely at genital level [13], although a possible association with intrauterine devices has been postulated. There have also been reports of its relationship with potentially fatal diseases such as Fournier’s gangrene [14]. In the present study, six of the eight cases (66.7%) of SVG isolates were symptomatic, and one had a tubo-ovarian abscess in the setting of PID. In male adults, most isolates in genital infections corresponded to *C. glucuronolyticum* (32.8%) or *Gardnerella* spp. (32.8%).

*Corynebacterium* spp. form part of normal skin and mucosa microbiota but can act as pathogens not only at genitourinary level but also in wound and soft tissue infections, endocarditis, bacteremia, and osteomyelitis [15]. At genital level, *C. glucurunolyticum* has been associated with bacteremia, prostatitis, urethritis, and encrusted cystitis [16]. Almost all cases in the present study corresponded to monomicrobial isolation (17/19; 89.5%), indicating its important pathogenic role, and this microorganism was also associated with cases of acute/chronic prostatitis.

*Gardnerella* spp., a genus of Gram-variable, facultative, anaerobic, immobile bacillus, were the second most frequent etiological agent in the present study, mainly in female patients (78.9%) [17]. Female genitalia are its main reservoir, and it is occasionally only a colonizer [18]; in this regard, new species were recently identified (*Gardnerella leopoldii* and *Gardnerella swidsinskii*) that form part of the female urinary microbiome alongside *G. vaginalis* [19]. However, this species are of microbiological and clinical relevance in certain circumstances and can possibly be associated with bacteremia, endocarditis, renal abscess, and urethritis [20]. It can also form part of the urogenital microbiota in 7–11% of male patients. In male adults, it was the second most frequent etiological agent at genital level but was concomitantly isolated with other microorganisms in 12/19 (63%). 

Other important microorganisms were less frequently isolated at genital level, including *Pasteurella bettyae*, which was only isolated in three samples, including two from patients with HIV; this species rarely produces disease in humans, although it has been associated with urethritis and balanitis [21] and can occasionally cause fatal lung disease [22]. Another microorganism, *E. corrodens*, is a part of female genital microbiota but has also been related to polymicrobial pelvic infections and chorioamnionitis. It was isolated in three endocervical samples from the present female adults, being isolated with concomitant isolates in two of these samples but alone in the third, which met criteria for PID, suggesting the need to consider its potential pathogenic role at this level [23]. Seven isolates of *F. hominis* were detected in the present series, including three related to immunosuppression, although five of them were interpreted as contamination. This species is rarely implicated in human infections but has been associated with sepsis, bacteremia, genitourinary infections [24,25] and even prosthetic infections [26]. *Moraxella osloensis* has rarely been implicated in genitourinary infections, but two isolates were detected at genital level in patients with permanent catheter [27]. *Leptotrichia* spp. corresponds to a genus of Gram-negative bacilli that are part of the oropharyngeal and genital microbiota but can produce bacteremia/sepsis and genitourinary symptoms in immunosuppressed individuals [28]. In fact, only one isolate in the present series (*L. trevisanii*) was clinically and analytically associated with PID [29]. Two isolates of *N. meningitidis* were detected, one in a sample from a HIV-positive male with urethritis; this microorganism is increasingly associated with urethritis and proctitis cases in men who have sex with men [30,31]. *Actinotignum* spp. and *Actinobaculum* spp. are genera of Gram-positive bacilli that can be responsible for urine infections [32], bacteremia [33], and endocarditis [34], mainly in elderly patients with chronic disease. At genital level, *Actinotignum* spp.has been described as a cause of balanitis and balanoposthitis [35]. Among the eight isolates detected in genital infections, two were from patients with pediatric balanoposthitis by *A. sanguinis* and *A. schaali* [36], respectively, and one from a patient with chronic prostatitis by *A. schaali* [37]. The high percentage of symptomatic episodes (6/8; 66.7%) and antibiotic prescriptions (66.7%) underscore the pathogenic role of this genus. *A. massiliense* can be part of the genitourinary microbiota and has numerous phylogenetic similarities with *Actinotignum* spp., and the sole isolate at genital level was in the setting of PID [38].

Among female adults with UTI, the most frequent emerging microorganisms were SBG (43.5%) and *Gardnerella* spp. (22.1%). Matesanz et al. described the pathogenic role in urinary infections of SBG [39], which was detected in 72 (70%) of the 91 females with urinary infection in the present study, mostly corresponding to the *S. gallolyticus* subspecies *pasteurianus* [40]. In our study, SBG was also associated with the presence of underlying conditions such as diabetes mellitus (13/91; 14.3%), solid organ transplantation (13/91; 14.3%), and metastatic solid neoplasm (12/91; 13.3%), as previously reported [41]. We highlight the detection of SBG in 100% of the 15 female children with UTI in this series.

*Gardnerella* spp. should be considered in cases of recurrent urinary infection with no detection of habitual pathogens, given their association with vesical microbiome disorders [42], and they are frequently isolated concomitantly with *E. coli*. In the present study, seven (18.4%) of the 38 patients with episodes due to *Gardnerella* spp. had some type of structural/anatomical urinary tract disorder. In the male adults, the commonest isolates at urinary level were SBG (23.3%), *A. urinae* (13.7%), and *Gardnerella* spp. (12.3%). Almost one-third of the males with infection by SBG had urinary tract disorders (6/19; 31.6%), whose clinical relevance should therefore be taken into account [43]. 

Urinary infections caused by *Aerococcus* spp. are often mild; however, the clinical significance of this genus is gradually increasing due to the risk of dissemination and complications [44] such as bacteremia and endocarditis [45]. The most frequent risk factors are higher age and the presence of urological disorders [46], and a statistically significant association was observed with the male sex and higher age (median of 77 yrs) in the present series. *Aerococcus* spp. was also involved in a higher percentage of symptomatic episodes in comparison to other genera and species, highlighting the need to interpret these isolates as pathogens and act accordingly. It is crucial to consider the possibility of complications and the age of patients, given that septic shock and death were recorded in 7.7% of the patients with infection by *Aerococcus* spp. A urinary tract disorder or permanent vesical catheter was present in 21% of cases. The most frequent species was *A. urinae*.

*Gardnerella* spp. are much less frequent etiological agents in urinary infections but must be considered in the presence of anatomical urinary tract disorders, when they can cause bacteremia and have important clinical consequences [47]. Among the nine males with *Gardnerella* spp. isolates in urine samples, seven (77.8%) were solid organ transplantation patients. Although most of the males had asymptomatic bacteriuria, many of them were screened and received antibiotic treatment. Screening of asymptomatic bacteriuria is not currently recommended in patients transplanted less than two months earlier due to the risk of infection by resistant bacteria [48].

In regard to *Corynebacterium* spp. [49], urinary infections by these species are associated with interactions with health care, immunosuppression, and the presence of chronic disease (e.g., chronic kidney disease) [50]. Indeed, more than 40% of infected patients in this study had some degree of immunosuppression, 18.2% had some association with healthcare, 27.3% were carriers of a permanent vesical catheter, and 41% had a history of antibiotherapy in the previous three months. More than 90% of patients with genital infections by these species were male, and there was a high percentage with chronic prostatitis. The most frequent species was *C. glucurunolyticum*. We highlight the need to consider this genus in cases of chronic prostatitis with no presence of typically responsible isolates.

Other microorganisms were isolated at urinary level in both sexes and should not be ignored, including: 

*Actynomices turicensis*, one of the species of this genus that has been related to genitourinary infections, with reports of its association with cystitis, balanitis, and prostatitis [51,52]. The four urinary isolates in the present study were all in samples from immunosuppressed patients (chronic kidney disease in hemodialysis and kidney transplantation) with no associated symptoms, so that treatment was not ordered by the clinician for half of these patients. However, the two-year-old girl with an isolate of endocervical origin was symptomatic and received treatment.

*Alloscardovia omnicolens* is a commensal of the oral cavity and gastrointestinal tract and has been isolated in urine, blood, and pulmonary and valvular abscesses. It has a controversial pathogenic role at urinary level [53], although cases of urinary infection and bacteremia have been reported [54]. One of the three isolates detected in the present series was the sole urinary isolate from a kidney transplantation patient with systemic and urinary symptoms, underscoring its clinical importance in immunosuppressed patients. Unlike the genital episodes produced by *Actinotignum* spp. and *Actinobaculum* spp., only one (14.3%) of the episodes of urinary infection was symptomatic and treatment was prescribed in three (42.9%), despite the immunosuppression present in five cases (5/7; 61.2%). They are therefore likely to be colonizers or causes of asymptomatic bacteriuria, although clinicians should take account of its known role at urinary level [55,56].

*Lactobacillus* spp. was one of the least frequent genera detected in the present series. This anaerobic Gram-positive bacillus can form part of the habitual urinary microbiota [57], although it can also act as an opportunistic pathogen in immunosuppressed patients, producing arthritis [58], liver abscess [59], cholecystitis, or urinary infection [60,61]. Clinicians must therefore be alert when *Lactobacillus* spp. is isolated in patients with some type of immunosuppression (e.g., for kidney transplantation). Only 1 genital isolate and 23 urinary isolates were recorded in the present study. Reference is made to “*L. gasseri*/*L. paragasseri*” because differentiation of these species is not possible using the usual methods and requires advanced techniques such as phenylalanyl-tRNA synthase alpha subunit (pheS) gene sequencing, fluorescent amplified fragment length polymorphism fingerprinting, and multilocus sequence typing [62]. *L. gasseri/L. paragasseri* was the most frequent isolate (10/24; 41.7%), and the patients were elderly (median age of 71 years) and predominantly female (18/23; 75%), observing immunosuppression in 4/24 (16.7%), permanent vesical catheter in 4/24 (16.7%), and recent antibiotic treatment (<3 months earlier) in 8/24 (33.3%). In addition, urine analyses revealed leukocyturia and/or nitrites in 16/18 (88.9%), urinary or associated systematic symptoms were observed in 13/24 (54.2%), and elevated CRP was recorded in 10/24 (41.7%), being responsible for the death of one patient. It therefore appears important to avoid the universal consideration of a monomicrobial urinary isolate of species of the genus *Lactobacillus* as a contaminant, especially in elderly symptomatic patients with permanent vesical catheter and/or previous antibiotic load.

The pathogenic role of SVG is more doubtful at urinary than genital level because its isolation may result from genital microbiota contamination. In this way, five (83.3%) of the six cases in the present series were asymptomatic and unlikely to be clinically relevant.

Study limitations includes its observational single-center design, with no control group. However, microbiological isolates were only reported when their count was significant in urine cultures and their presence was abundant in genital exudates in order to enhance the robustness of results. In addition, analytical and clinical data, clinical outcomes, and the therapeutic approach of attending physicians were all considered in evaluating whether isolates play a pathogenic role or are microbiota members. 

## 5. Conclusions

Emerging microorganisms play an increasingly important etiological role in genitourinary infections, mainly detected when cultures are negative for the usual etiological agents. The clinical characteristics of patients should be taken into account, including their age, the presence of diabetes mellitus, immunosuppression, and/or permanent vesical catheter, and their previous antibiotic load. Urinary infections by *Aerococcus* spp. should be considered in males of advanced age, *Corynebacterium* spp. in male carriers of permanent vesical catheter, and SBG in female children. The presence of *Gardnerella* spp. as asymptomatic bacteriuria is frequent in kidney transplantation patients and those receiving chronic corticosteroid therapy. A single isolate of *Lactobacillus* spp. in urine should not be interpreted as a contaminant, especially in symptomatic patients with permanent vesical catheter and/or previous antibiotherapy. *Gardnerella* spp. should be considered as a possible cause of genital infection in patients with a history of risky sexual relations when microorganisms usually responsible for sexually transmitted infections are not detected.

## Figures and Tables

**Table 1:** Epidemiological and clinical variables corresponding to genital infection episodes in female adults.

Immunosuppression	Pregnancy	Previous Risky Sexual Relations	Concomitant Isolates	Associated Symptoms	Dysuria	Fever	Abdominal Pain	Leucorrhea	Vaginal Pruritus	Adenopathies	Pelvic Inflammatory Disease (PID)	Empirical Antibiotic Therapy	Recurrence
1/22 (4.5%)	3/22 (13.6%)	5/22 (22.7%)	9/22 (40.9%)	16/22 (72.7%)	5/22 (22.7%)	7/22 (31.8%)	10/22 (45.5%)	12/22 (54.5%)	3/22 (13.6%)	1/22 (4.5%)	4/22 (18.2%)	19/22 (86.4%)	1/22 (4.5%)

**Table 2:** Epidemiological and clinical variables corresponding to genital infection episodes in male adults.

Immunosuppression	Benign Prostatic Hyperplasia (BPH)	Prostatitis	Previous Risky Sexual relations	Concomitant Isolates	Associated Symptoms	Balanitis	Dysuria	Fever	Abdominal Pain	Rectal Tenesmus	Urethral Exudate	Adenopathies	Hemospermia	Empirical Antibiotic Therapy	Recurrence
8/58 (13.8%)	10/58 (17.2%)	Acute: 5/58 (8.6%) Chronic: 9/58 (15.5%)	20/58 (34.5%)	18/58 (31%)	40/58 (69%)	5/58 (8.6%)	21/58 (36.2%)	3/58 (5.2%)	23/58 (39.7%)	1/58 (1.7%)	6/58 (10.3%)	1/58 (1.7%)	5/58 (8.6%)	40/58 (69%)	5/58 (8.6%)

**Table 3:** Epidemiological and clinical variables corresponding to urinary infection episodes in male and female adults.

Sex	Immunosuppression	Solid Organ Transplantation	Chronic Corticoid Consumption	Anatomical Urinary Tract Disorder	Permanent Vesical Catheter	Diabetes	Association with Healthcare	Antibiotic Consumption (in Previous 3 Months)	Associated Symptoms	Dysuria	Fever	Abdominal Pain	Pathological Urine Analysis	Leukocytosis >12,000/mm^3^	Elevated C Reactive Protein	Empirical Antibiotic Therapy	Recurrence	Deaths
Female adults	68/131 (51.9%)	44/131 (33.6%)	45/131 (34.4%)	14/131 (10.7%)	9/131 (6.9%)	21/131 (16%)	17/131 (13%)	23/131 (17.6%)	66/131 (50.4%)	18/131 (13.8%)	45/131 (34.4%)	27/131 (20.6%)	72/92 (78.3%)	29/131 (22.1%)	40/54 (74.1%)	77/131 (58.8%)	3/131 (2.3%)	4/131 (3%)
Male adults	31/73 (42.5%)	24/73 (32.9%)	21/73 (28.8%)	17/73 (23.3%)	10/73 (13.7%)	14/73 (19.2%)	5/73 (6.8%)	13/73 (17.8%)	35/73 (47.9%)	14/73 (19.2%)	17/73 (23.3%)	19/73 (26%)	36/50 (72%)	19/73 (26%)	26/33 (78.8%)	49/73 (67.1%)	0/73 (0%)	2/73 (2.7%)

## Data Availability

The data presented in this study are available in the main text.

## Supplementary Materials

The following supporting information can be downloaded at: https://www.mdpi.com/article/10.3390/microorganisms11040915/s1, Table S1: list of episodes of genital and urinary infections and results for the study variables; Table S2: Isolated microorganisms and their frequency among genital infections in female adults; Table S3: Isolated microorganisms and their frequency among genital infections in male adults; Table S4: Isolated microorganisms and their frequency among urinary infections in female adults; Table S5: Isolated microorganisms and their frequency among urinary infections in male adults.
