# Supplementary material for: Presence and Relevance of Emerging Microorganisms in Clinical Genitourinary Samples
Source: Microorganisms. 2023 Mar 31;11(4):915. doi: 10.3390/microorganisms11040915 (PMC10146931; doi:10.3390/microorganisms11040915)
Supplement: Supplementary file 1 [file microorganisms-11-00915-s001.zip › microorganisms-2239092-Table S2-S5.pdf]

Supplementary Table S2. Isolated microorganisms and their frequency among genital infections in female adults.

Microorganism	SVG	*E. corrodens*	*Gardnerella* spp.	*Alloscardovia omnicolens*	*N. meningitidis*	*M. osloensis*	*P. bettyae*	*F. hominis*	*A. massiliense*	*A. christenseni*	*L. trevisanii*	*S. pneumoniae*	Total
Female	(7/22; 31.8%)	(3/22; 13.6%)	(2/22; 9.1%)	(2/22; 9.1%)	(1/22; 4.5%)	(1/22; 4.5%)	(1/22; 4.5%)	(1/22; 4.5%)	(1/22; 4.5%)	(1/22; 4.5%)	(1/22; 4.5%)	(1/22; 4.5%)	22 (100%)

Supplementary Table S3. Isolated microorganisms and their frequency among genital infections in male adults.

Microorganism	*C. glucuronolyticum*	*Gardnerella* spp.	SBG	*A. schaali*	*F. hominis*	*A. urinae*	*P. bettyae*	*A. sanguinis*	*A. sanguinicola*	*C. urealyticum*	*C. amycolatum*	*L. iners*	*M. osloensis*	*N. meningitidis*	Total
Male	(19/58; 32.8%)	(19/58; 32.8%)	(3/58; 5.2%)	(3/58; 5.2%)	(3/58; 5.2%)	(2/58; 3.4%)	(2/58; 3.4%)	(1/58; 1.7%)	(1/58; 1.7%)	(1/58; 1.7%)	(1/58; 1.7%)	(1/58; 1.7%)	(1/58; 1.7%)	(1/58; 1.7%)	58 (100%)

Supplementary Table S4. Isolated microorganisms and their frequency among urinary infections in female adults..

Micr.	SBG	*Gardnerella* spp.	*A. urinae*	*Lactobacillus*	*A. sanguinicola*	*Corynebacterium*	*A. schaali*	SVG	*A. massiliense*	*A. viridans*	*A. omnicolens*	*C. glucuronolyticum*	*C. jeikeum*	*C. minutissimum*	*C. urealyticum*	*F. hominis*	Total
Female	(57/131; 43.5%)	(29/131; 22.1%)	(7/131; 5.3%)	*gasseri/paragasseri: (6/131; 4.6%)* *jensenii: (4/131; 3%)* *crispatus: (3/131; 2.3%)* *delbrueckii: (3/131; 2.3%)* *fermentum:* (1/131; 0.7%), *rhamnosus: (1/131; 0.7%).*	(4/131; 3%)	*amycolatum* (4/131; 3%) *glucuronolyticum* (1/131; 0.7%) *jeikeum* (1/131; 0.7%) *minutissimum* (1/131; 0.7%) *urealyticum* (1/131; 0.7%)	(2/131; 1.5%)	(2/131; 1.5%)	(1/131; 0.7%)	(1/131; 0.7%)	(1/131; 0.7%)	(1/131; 0.7%)	(1/131; 0.7%)	(1/131; 0.7%)	(1/131; 0.7%)	(1/131; 0.7%)	131 (100%)

Supplementary Table S5. Isolated microorganisms and their frequency among urinary infections in male adults.

Micr.	SBG	*A. urinae*	*Gardnerella* spp.	*A. sanguinicola*	*Corynebacterium*	*Lactobacillus*	SVG	*A. turicensis*	*A. schaali*	*A, massiliense*	*F. hominis*	Total
Male	(17/73; 23.3%)	(10/73; 13.7%)	(9/73; 12.3%)	(5/73; 6.8%)	*glucuronolyticum* (5/73; 6.8%)<br>*urealyticum* (5/73; 6.8%)<br>*aurimucosum* (1/73; 1.4%)<br>*jeikeum* (1/73; 1.4%)<br>*striatum* (2/73; 2.7%)	*gasseri/paragasseri* (4/73; 5.5%)<br>*rhamnosus* (1/73; 1.4%)	(4/73; 5.5%)	(4/73; 5.5%)	(2/73; 2.7%)	(2/73; 2.7%)	(1/73; 1.4%)	73 (100%)
